# Large-scale manufacturing of immunosuppressive extracellular vesicles for human clinical trials

**DOI:** 10.1016/j.jcyt.2025.06.003

**Published:** 2025-06-13

**Authors:** Meizhang Li, Rupal Soder, Sunil Abhyankar, Trisha Home, Harsh Pathak, Xingqi Shen, Andrew K. Godwin, Haitham Abdelhakim

**Affiliations:** 1 Department of Pathology and Laboratory Medicine, University of Kansas Medical Center, Kansas City, Kansas, USA; 2 Midwest Stem Cell Therapy Center, University of Kansas Medical Center, Kansas City, Kansas, USA; 3 Division of Hematologic Malignancies and Cellular, Therapeutics, University of Kansas Medical Center, Kansas City, Kansas, USA; 4 The University of Kansas Cancer Center, University of Kansas Medical Center, Kansas City, Kansas, USA; 5 Department of Oncology, McArdle Laboratory of Cancer Research, University of Wisconsin–Madison, Madison, Wisconsin, USA; 6 Kansas Institute for Precision Medicine, University of Kansas Medical Center, Kansas City, Kansas, USA

**Keywords:** extracellular vesicles, large-scale manufacture, PD-L1, SEC, TFF, WJMSCs

## Abstract

**Background:**

Human mesenchymal stromal cells (MSCs), particularly Wharton’s jelly-derived MSCs (WJMSCs), offer significant therapeutic potential for complex immune conditions such as graft versus host disease (GVHD), in part through their secreted small extracellular vesicles (sEVs). These sEVs exhibit crucial immunomodulatory properties, including suppression of T-cell activation demonstrated both in healthy donor cells and in pathologic contexts. Despite this promise, widespread clinical application is impeded by substantial challenges in developing robust, scalable, and Good Manufacturing Practice (GMP)–compliant manufacturing processes for MSC-derived sEVs to meet clinical demand.

**Methods:**

To address the critical barriers in sEV production, this study details the development and validation of a reliable and scalable manufacturing platform for WJMSC-derived sEVs. The process utilized GMP expanded WJMSC culture medium, generated from cultured low-passage cells and processed in scalable 2-L and 6-L batch volumes. This platform employed a sequential approach involving tangential flow filtration (TFF) for efficient initial concentration followed by size-exclusion chromatography (SEC) for comprehensive final purification of sEVs.

**Results:**

The integrated TFF-SEC manufacturing approach resulted in a significant enrichment of nanoparticles, demonstrating up to a 16.9- and 36-fold increase in particle concentration post-TFF from the 2-L and 6-L batches, respectively. Two batches of purified sEVs demonstrated very similar mean size ranges, from 142 ± 2 nm to 156 ± 2 nm, and displayed the markers CD9 and CD81 while not expressing the negative marker calnexin. Those WJMSC-derived sEVs maintained their biological activity, effectively suppressing - cell activation *in vitro*. Furthermore, the purified sEVs from both 2-L and 6-L batches demonstrated an intact structure observed by cryogenic electron microscopy (cryo-EM), positivity for the inhibitory immune checkpoint ligand PD-L1.

**Conclusion:**

In this study, we report a reliable large-scale manufacturing framework that combines TFF and SEC to manufacture WJMSC-derived sEVs. The established standard operating procedures (SOPs) will help guide the design and establishment of industrial-scale, clinical-grade WJMSC-derived sEV manufacturing. This work significantly advances the field by offering a practical pathway that is anticipated to facilitate the broader development and accelerate the clinical translation of these WJMSC-derived sEVs as potent, cell-free therapeutic agents for various human diseases.

## Introduction

Small extracellular vesicles (sEVs) are secreted nanoparticles generally smaller than 200 nm and are usually involved in fundamental communications with other types of cells or are progressively modifying the surrounding tissue microenvironment [1–3]. For example, immune checkpoint PD-L1 from tumor-derived sEVs functionally exhausts the activated CD8^+^ T cells, leading to the escape of tumor cells from immune surveillance [4,5]. Mesenchymal stromal cells (MSCs) secrete sEVs that are loaded with similar immune regulators as their parental cells and are capable of suppressing T cells [6–8]. MSCs can decrease cellular proliferation and T-cell activation through the PD-L1 signaling pathway in patients with acute graft versus host disease (aGVHD) [9]. In a recent clinical trial at our center, we found that Wharton’s jelly-derived MSCs (WJMSCs) therapy was safe and potentially effective in treating patients with aGVHD [10]. We reported that elevated levels of PD-L1 detected on sEVs in plasma from aGVHD patients are directly associated with WJMSC infusion [11]. Moreover, WJMSC-derived sEVs lost their immune suppressive potential after genetic disruption of the *PDL1* gene in their host cells, supporting the rationale for developing sEV-based therapies for immune diseases [12]. These findings support the rationale for isolating highly purified immune suppressive WJMSC-derived sEVs and developing sEV-based cell-free therapies for aGVHD.

The industrial manufacturing of therapeutic sEVs currently has many obstacles that need to be thoroughly evaluated, including tissue sources, cell culture, EV engineering, large-scale processes, and long-term storage aspects [13–15]. There is currently no consensus regarding a standardized framework for isolation of sEVs for clinical use [16,17]. Despite the improvements in 3D culture from bioreactors [18,19], genetic engineering [20,21], and chemically defined medium formulation [22,23], multiple scalable conditional medium processing protocols have been suggested for the industrial field to harvest high-grade sEVs [24,25]. Börger et al. reported the development of a polyethylene glycol (PEG)–based precipitation protocol allowing extraction of EVs from several liters of conditioned medium from MSC culture [26]. However, drawbacks such as a lack of closed-loop conditions and mid-range scalability can limit wide-scale applications. As an efficient ultrafiltration system, tangential flow filtration (TFF) has also been employed for large-scale sEVs production [27–30]. TFF has demonstrated suitability for different processes that meet the requirements of Good Manufacturing Practice (GMP) [27,31]. More importantly, combined with size-exclusion chromatography (SEC), the TFF-based platform will have more advantages for producing clinical-grade sEVs with higher purity [27,32]. However, this combined platform has not been reported yet for immunosuppressive WJMSC-derived sEVs. In this study, we explored the large-scale production of clinical-grade WJMSC-derived sEVs using this combined strategy of SEC and TFF. We compared the yield and the quality of WJMSC sEVs produced from low-volume (2 L) and high-volume (6 L) batches of WJMSC GMP-compliant culture medium.

## Materials and Methods

### Production of WJMSC culture medium under GMP guidelines

A de-identified umbilical cord tissue sample was obtained from a donor enrolled under the Institutional Review Board-approved protocol (HSC#1546) following GMP as described before [10]. Briefly, an umbilical cord was collected from a healthy adult woman who underwent elective cesarean section after a full-term pregnancy. After thoroughly washing the cords twice with phosphate buffered saline (PBS), blood vessels within the tissues were carefully and completely removed. The cord tissues were dissociated into small pieces around 2 to 3 mm in diameter. Dissociated tissues were further seeded onto culture dishes containing xeno-free culture media, MSC Nutristem XF Basal Medium (Sartorius, Puerto Rico, USA), supplemented with MSC NutriStem XF Supplement (Sartorius) and PLTGold Human Platelet Lysate (PLT Gold Clinical Grade), penicillin 100 U/mL, and streptomycin 100 *μ*g/mL (Mediatech). Explant cultures were incubated at 37°C in a humidified atmosphere containing 5% CO_2_. Medium was changed every 3 to 4 days and tissue explants were removed after 21 days of culture to allow the migration of cells from the explants. Once between 80% and 90% confluency, adherent cells were trypsinized using TrypLE Select (Life Technologies) and reseeded in tissue culture flasks (Corning) for further culture expansion. Low-passage WJMSCs (<4 frozen/thawed cycles) were cultured in a 175 cm^2^ flask using 50 mL of the same culture medium described above. To harvest sEVs, WJMSCs (passage 4) were cultured in 1720 cm^2^ Corning CellBIND Surface HYPERFlask cell culture vessels (Millipore, USA) using a 550-mL culture medium. On day 6, all media were collected once and long-term stored at −80°C before any further application.

### Bicinchoninic acid (BCA) assay

Protein quantitation was carried out using the Micro BCA Protein Assay Kit (Thermo Scientific, USA) following the protocol provided by the manufacturer. Briefly, 10 *μ*L of samples was diluted into 40 *μ*L of ddH_2_O, mixed with 50 *μ*L of BCA regents. All samples were loaded on a 96-well plate and incubated for 1 hour at 37°C. Optical density (OD) value was read using the Infinite 200 PRO plate reader (Tecan, USA) at 562 nm wavelength.

### Tangential flow filtration (TFF)

The stored frozen culture medium was thawed and precleaned using a Sorvall Lynx 6000 centrifuge. The culture medium was spun for 30 minutes at 3000 rpm to remove cellular debris, followed by two 60-minute runs of centrifugation at 10,000 g to remove macrovesicles. The precleaned medium was immediately processed using a Smartflow Smart TFF system (Sartorius, NY, USA). Sartocon Slice Cassettes with Polyethersulfon membrane and 0.02~0.14 effective filtration area (m^2^) and 300 KD cut-off (item number: 3M81467902E-SW and 3M51467901E-SW, Sartorius) were applied to concentrate the culture medium. The media were initially concentrated into 30–100 mL of volume according to the tested parameters provided in Supplementary Table 1. Diafiltration was performed at a concentration factor of 30 to 60 using exosome-free PBS and the number of diavolumes was between 8 and 10.

### Size-exclusion chromatography (SEC)

Extracellular vesicles were purified using commercial EX04 Midi resin columns (Cell Guidance Systems Ltd, USA), containing pores with a diameter of approximately 30 nm. Columns were washed with 5 to 10 volumes of EV-free PBS before use. In brief, we manually loaded 1 mL of TFF-concentrated samples into the column and collected all flow fluid in an Eppendorf tube. Next, 1 mL of sEV-free PBS was added to the column, and all flow fluid was collected in another Eppendorf tube. We repeated this step until we collected 12 elution samples. Flow fluid rates were 4 to 7 drops/minute, 40 to 45 mL per drop.

### Nanoparticle tracking analysis (NTA)

Nanoparticle tracking analysis (NTA) was performed using the NanoSight LM10 system (NanoSight Ltd; United Kingdom) following MISEV guidelines [3]. Particles were diluted into EV-free PBS by 1000- to 40,000-fold. Diluted particles (1 mL) were injected into the chamber at a syringe speed of 40 *μ*L/s. Both particle number and size of WJMSC sEV were collected and analyzed using NTA software v2.3 (NanoSight Ltd). For each measurement, five ~1-minute videos and ~1498 frames were captured under the cell temperature of 22.4°C. Equipment settings included Brightness = 0; Gain = 1; Detection threshold = 10, Min track length = 10 steps. Measurement conditions included Camera shutter 9 (ms) = 11.97; Camera gain = 350, Frame rate (fps) = 9.79; Camera bit-conversion limits = 0; Viscosity (cP) =1.01.

### Automated western assay (WES)

WES was carried out according to the standard procedures provided by the manufacturer (Bio-Techne, MN, USA). Briefly, samples were diluted with 0.1 X Sample Buffer to 0.4 mg/mL. Four parts of the prepared lysate were mixed with 1 part of the 5 X Fluorescent Master mix (containing the reducing agent DTT). After mixing gently with a pipette, the samples were vortexed and heated at 95°C for 5 minutes to denature them. The samples were vortexed again, spun down briefly, and stored on ice. Then, samples and other WES regents were loaded onto the plate (supplied with the kit) using the recommended volumes as per protocol. The plate and the Capillary Cartridge were then carefully loaded into their respective positions in the WES Protein Simple instrument. WES primary antibodies of purified rabbit monoclonal antibodies anti-human CD81 (R&D Systems, USA) and anti-human CD9 (Cell Signaling Technology, USA), mouse antibodies anti-human CD73, albumin and calnexin (R&D Systems), and of purified mouse anti-human PD-L1 and isotype control (BioLegend, USA). Horseradish peroxidase (HRP)–conjugated secondary antibodies were purchased from Bio-Techne (USA).

### Flow cytometry

To make CD9-positive beads, a mouse anti-human CD9 antibody (BioLegend) was conjugated to NHS Act Sepharose 4 Fast Flow (Millipore Sigma, USA) following the protocols provided by the manufacturer. Extracellular vesicles were captured by CD9-positive beads and further stained with PE-conjugated anti-human PD-L1 (BioLegend). Human peripheral blood mononuclear cells (PBMCs) were stained by anti-human antibodies: anti-CD3 PE-Cy5/CD4 PE/CD8 FITC Cocktail and APC anti-CD154. All antibodies were used at a dilution of 1:100. Flow cytometric analysis was performed using the BD LSR II analyzer (Becton Dickinson, USA) or the Attune NxT multiparameter flow cytometer (Invitrogen, USA).

### Transmission Electron Microscopy (TEM)

TEM was carried out as the standard protocol. In brief, 10 *μ*L of isolated sEVs were dropped on the spot plate and glow discharge carbon filmed nickel grids (Electron Microscopy Sciences, USA) were floated on the drops for 20 minutes. Grids were washed 3 times with H_2_O and fixed with 2.5 glutaraldehyde in 100 mM sodium cacodylate buffer (pH 7.0) for 1 hour. Negative staining was performed by using a 3% solution of neutral sodium phosphotungstate for 20 seconds. Grids were washed 3 times with H_2_O. Finally, grids were fixed with 1% glutaraldehyde and stained with 1% uranyl acetate for 5 seconds, dried, and viewed under a JEOL JEM-1400 transmission electron microscope (JEOL, USA) equipped with a Lab6 gun at 100 kV.

### Cryogenic electron microscopy (cryo-EM)

Quantifoil 300 mesh R1.2/1.3 holey carbon over copper substrate grids was prepared by glow discharging using a Pelco easiGlo glow discharge unit set to protocol 3 for 3 cycles × 45 seconds. Using a TFS Vitrobot Mark IV, samples at a concentration of 1.3 mg/mL were added to glow discharged grids at 4°C and 100% humidity and underwent a double application and blotting procedure as follows: First, sample application of 3 mL with a wait time of 20 seconds followed by a 1-second blot time at a blot force of 0; second, sample application of 3 mL with a 5-second wait time followed by a 3-second blot at a blot force of 5. Grids were then rapidly plunged frozen into liquid ethane at a temperature of −184 °C. Images were collected on a TFS Glacios 200 kV TEM equipped with a Falcon 4i Direct Electron Detector and Selectris energy filter. All images were collected at a nominal magnification of 100,000 in EER counting mode with a binning factor of 1 and Selectris slit width of 10 eV.

### Enzyme-linked immunosorbent assay (ELISA)

Samples were added to a 96-well plate to detect human type I collagen using the ELISA kit according to the manufacturer’s instructions (AFG Bioscience, USA). OD value was read at an absorbance of 450 nm using the Infinite 200 PRO plate reader (Tecan, USA).

### *In vitro* inhibition of T-cell activation

We used 5 mL whole blood to prepare PBMCs using lymphocyte separation medium (Corning, USA) and spun for 30 minutes at 400 × g through density gradient centrifugation. After washing with PBS, 1 × 10^5^ PBMCs per well were seeded into 96-well plates with 1 mL of Hyclone RPMI-1640 medium (GE Healthcare Life Sciences, USA) supplemented with 10% FBS, 10 *μ*M HEPES buffer, and 100 U/mL penicillin-streptomycin. PBMCs were activated with CD3/CD28 Dynabeads (Gibco, USA) at a dilution of 1:1 ratio as previously described [39]. Stimulated PBMCs were further treated with WJMSC sEVs at a concentration of 8 × 10^3^ particles/cell for 12 hours. T-cell activation was measured by dual CD154^+^/CD4^+^ with flow cytometry (as described previously) [11].

### Statistics

Data were analyzed and statistics were performed in GraphPad Prism 8. Data were expressed as means ± SEM (standard error of the mean). A one-tailed, unpaired Student’s *t*-test was used for pair-wise comparison or one-way analysis of variance (ANOVA) with Dunnett’s test. *P < 0.05, **P < 0.01, ***P < 0.005, and ****P < 0.001.

## Results

### Large-scale production of GMP-compliant WJMSC culture medium

**Figure 1.**
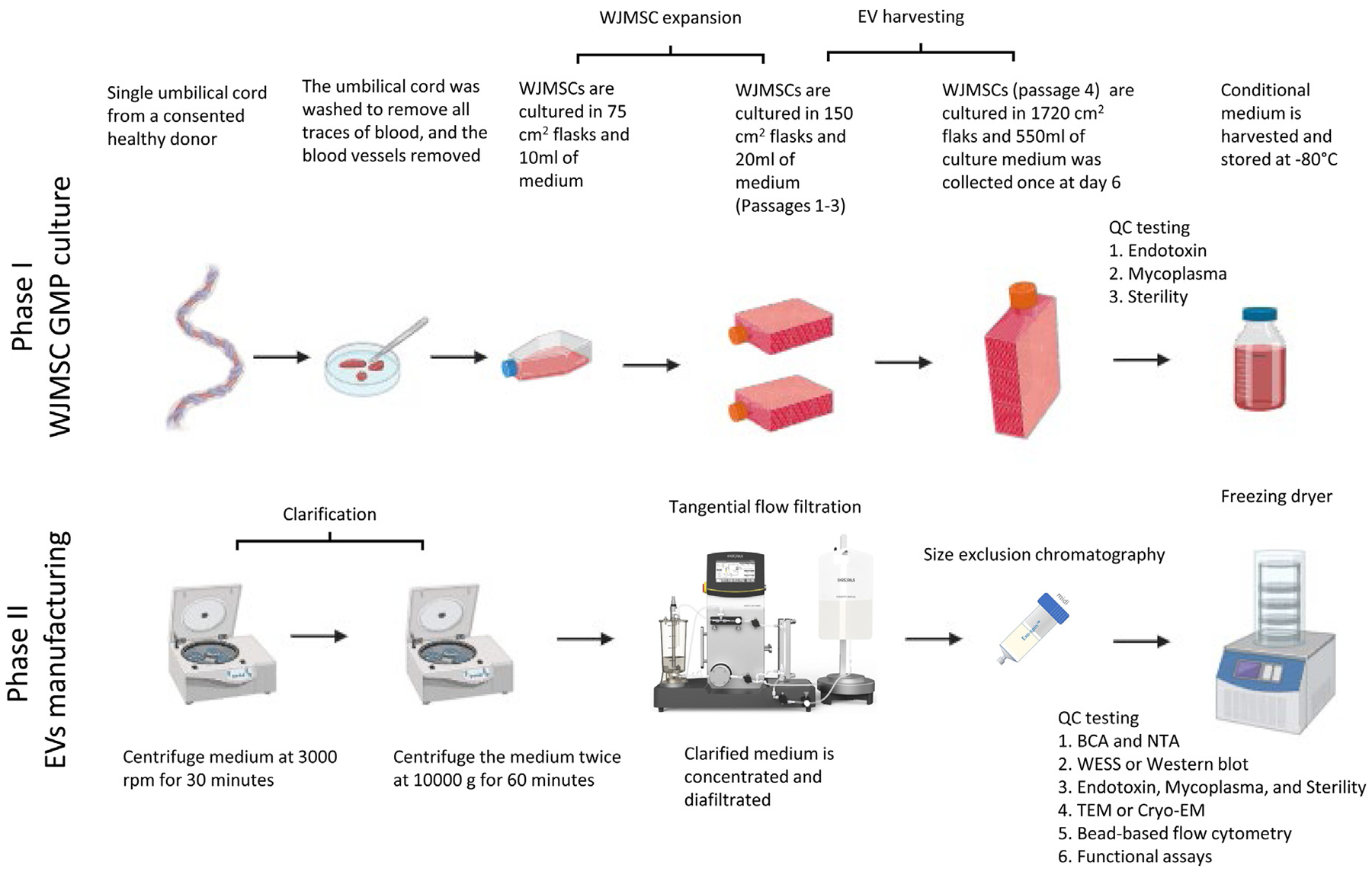
The strategy of large-scale extracellular vesicle (EV) manufacture. The diagram demonstrates the standard operating procedures (SOPs) for therapeutic EV manufacturing. (A) Phase I: Wharton’s jelly–derived mesenchymal stromal cells (WJMSC) conditional culture medium (CM) was first produced through good manufacturing practice (GMP) at the Midwest Stem Cell Therapy Center (MSCTC). (B) Phase II: The precleaned conditional medium was next concentrated using tangential flow filtration (TFF) with a 300-KD cutoff. Size exclusion chromatography (SEC) was further applied to isolate WJMSC-derived small extracellular vesicles (sEVs). Finally, WJMSC-derived sEVs were lyophilized using a freeze dryer and stored at −80°C refrigerator for long-term storage.

The large-scale manufacturing of WJMSC cell culture medium was completed by the GMP facility of the Midwest Stem Cell Therapeutic Center (MSCTC) at the University of Kansas Medical School utilizing an umbilical cord tissue sample (Figure 1A). All media were immediately frozen and stored at the −20°C refrigerator after passing the validations of endotoxin, mycoplasma, and sterility following the requirements of good quality controls according to the GMP-compliant regulations (Table 1).

### Small-scale concentration of WJMSC culture medium

**Figure 2.**
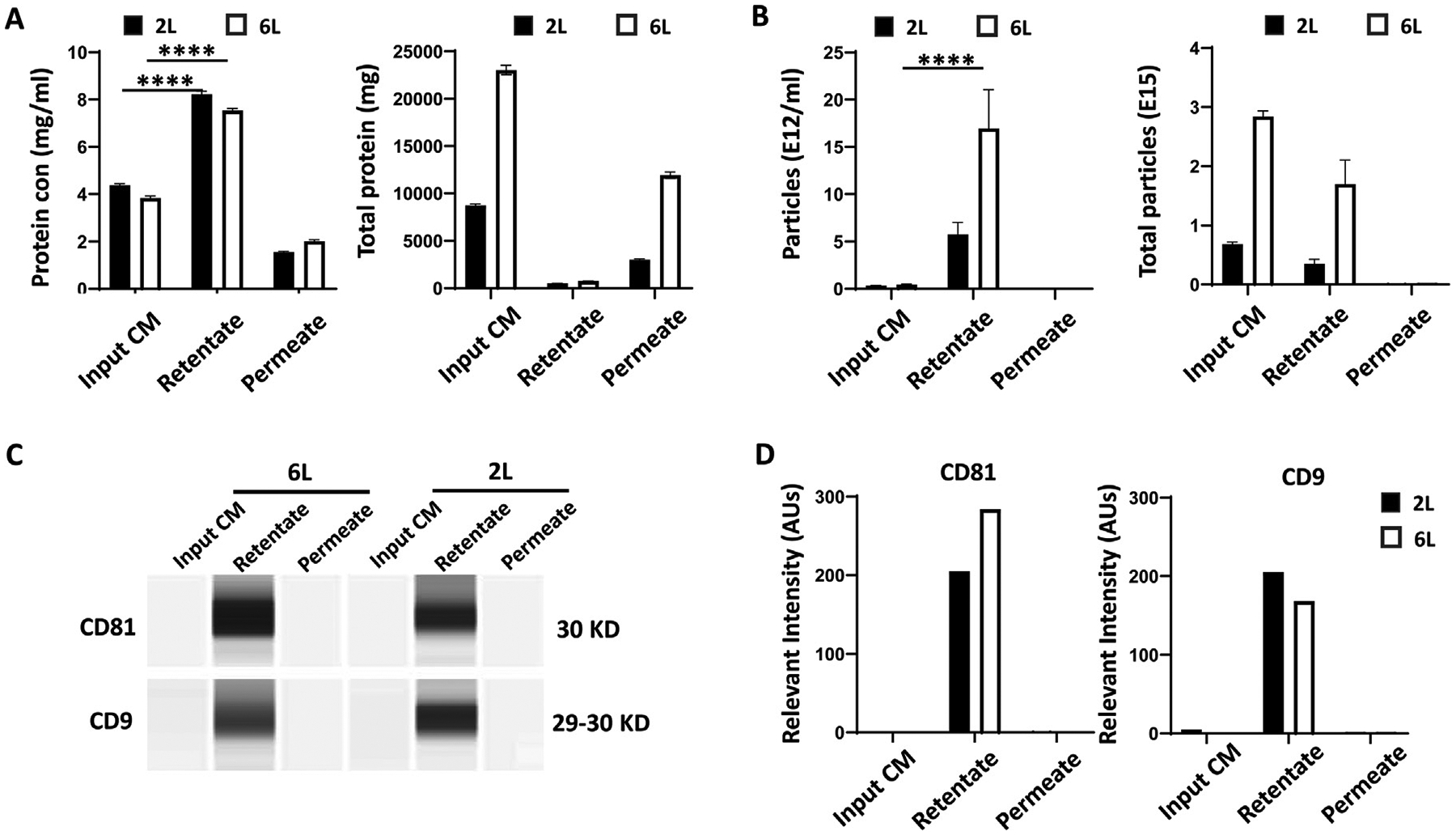
Tangential flow filtration (TFF)–based concentration of good manufacturing practice (GMP)–compliant Wharton’s jelly-derived mesenchymal stromal cell (WJMSC) conditioned medium (CM). WJMSC culture media (2 L and 6 L) were individually concentrated by TFF with a 300-KD cutoff. (A) The total and average protein measurements of input CM (original medium), retentate, and permeate were analyzed by bicinchoninic acid (BCA) assay. Total protein (*left*) and protein concentration per milliliter (*right*). (B) The total and average particle numbers from (A) were measured by nanoparticle tracking analysis (NTA). Total particles (*left*) and particle concentration per milliliter (*right*). (C, D) Protein detection of extracellular vesicle biomarkers CD9 and CD81 from (A) using simple western (WES). Representative western imaging (C) and relevant intensity of western bands (D). Values on graphs represent means ± SEM, n = 3 individual measurements. ****P < 0.001 (A, B).

We completely thawed and centrifuged 2 L of WJMSC culture medium to clear cell debris and other macro extracellular vesicles. The processed medium was further concentrated by TFF with a 300-KD molecular weight cutoff (Figure 1B). A 61-mL final concentrated sample was generated from the retentate of TFF-based filtration. We detected 8.2 ± 0.07 mg/mL and total 501 mg of protein from the retentate compared with 4.4 ± 0.04 mg/mL and total 8750 mg protein from the original 2-L input medium (Figure 2A). There is about 94% of loss of total protein in the retentate (61 mL) from input medium (2 L), indicating the effective removal of soluble proteins from the retentate. To determine the concentration effect for particles, we applied NTA to measure the particle number; 5.75 ± 1.3 × 10^12^/mL or 1.6 × 10^11^/mg of particle concentration was detected in the retentate, which is a 16.9-fold increase when compared with 0.34 ± 0.015 × 10^12^/mL of particle concentration in the original input medium (Figure 2B). When we compared the total particles of both input medium (2 L) and the retentate part (61 mL), about 49% of particles were recovered after 1 cycle of concentration and diafiltration (Figure 2B). Particles from the retentate part showed an average 78 ± 7 nm mode size and 102 ± 4 nm mean size, which were not different from the original input medium (Supplementary Figure 1). To confirm these observations, antibodies anti-human CD9 and CD81 were further used to examine the enrichment of sEVs in those samples. Consistently, both sEV biomarkers demonstrated a higher protein level in the retentate medium than in the original input medium (Figure 2C,D). These results demonstrate that TFF-based concentration efficiently enriches nanoparticles from the GMP-compliant WJMSC culture medium.

To our surprise, few nanoparticles were observed from the permeate part although 2 ± 0.03 mg/mL of the total protein was still detectable from the same samples (Figure 2A,B). This finding supports the fact that abundant small soluble protein (<300 KD molecular weight) had been successfully separated from the nanoparticles at this stage. Neither human CD9 nor CD81 was detected by simple western (WES) in the permeate part (Figure 2C,D). To investigate the potential particle contamination from human platelet lysates, we further conducted another comparative TFF concentration using 2 L of basal medium (without WJMSC culture) and conditioned medium (with WJMSC cell culture). After TFF concentration, ~1 ± 0.11 × 10^14^ total particles were detected in the retentate part (28 mL) of the conditional medium (Supplementary Figure 2C). However, only 0.19 ± 0.01 ×10^14^ total particles were found in the retentate part (30 mL) of basal medium, indicating <20% potential particle contamination contribution by human platelet lysate supplement in the medium. In addition, we applied ELISA to examine the protein level of human type I collagen, one of the abundant collagen proteins from the umbilical cord tissue. In the retentate (concentrated) part, no type I collagen was detected (Supplemental Figure 3). Our results showed that molecular weight cutoff below 300 KD from the TFF was sufficient to concentrate sEV-sized nanoparticles (40–150 nm).

### Large-scale concentration of WJMSC culture medium

To test the enlarging capacity and efficiency of our TFF platform, we further scaled up processing the 6 L of WJMSC culture medium (Figure 1B). A 100-mL final concentrated sample was generated from the retentate part of TFF-based filtration. We observed 7.54 ± 0.05 mg/mL and total 753 mg of protein from the retentate part (Figure 2A). We detected 3.84 ± 0.05 mg/mL and total 8750 mg protein from the original input medium (6 L). About 97% of loss of total protein was noticed when compared with the input medium (6 L) and the retentate (100 mL). We detected 17 ± 4.1 × 10^12^/mL or 2.25 × 10^12^/mg of particle concentration from the retentate sample and nearly increased by 36-fold when compared with 0.47 ± 0.015 × 10^12^/mL of particle concentration from the original input medium (Figure 2B, *left*). About 40% of particles were concentrated after 1 cycle of concentration and diafiltration (Figure 2B, *right*). Comparing small-scale (2 L) and large-scale (6 L) processing, isolated nanoparticles demonstrated very similar mode sizes and mean sizes by NTA analysis (Supplementary Figure 1). Furthermore, we observed that CD9 and CD81 were enriched in the retentate part from both processing (Figure 2C,D). Again, no type I collagen was found in the retentate parts of both processing methods (Supplementary Figure 3), indicating filtering out of soluble proteins. Therefore, current studies support that our TFF-based concentration provides a simple but very efficient platform to process the large-scale WJMSC culture medium and significantly improve the final yield of nanoparticles.

### Purification of WJMSC-derived sEVs

**Figure 3.**
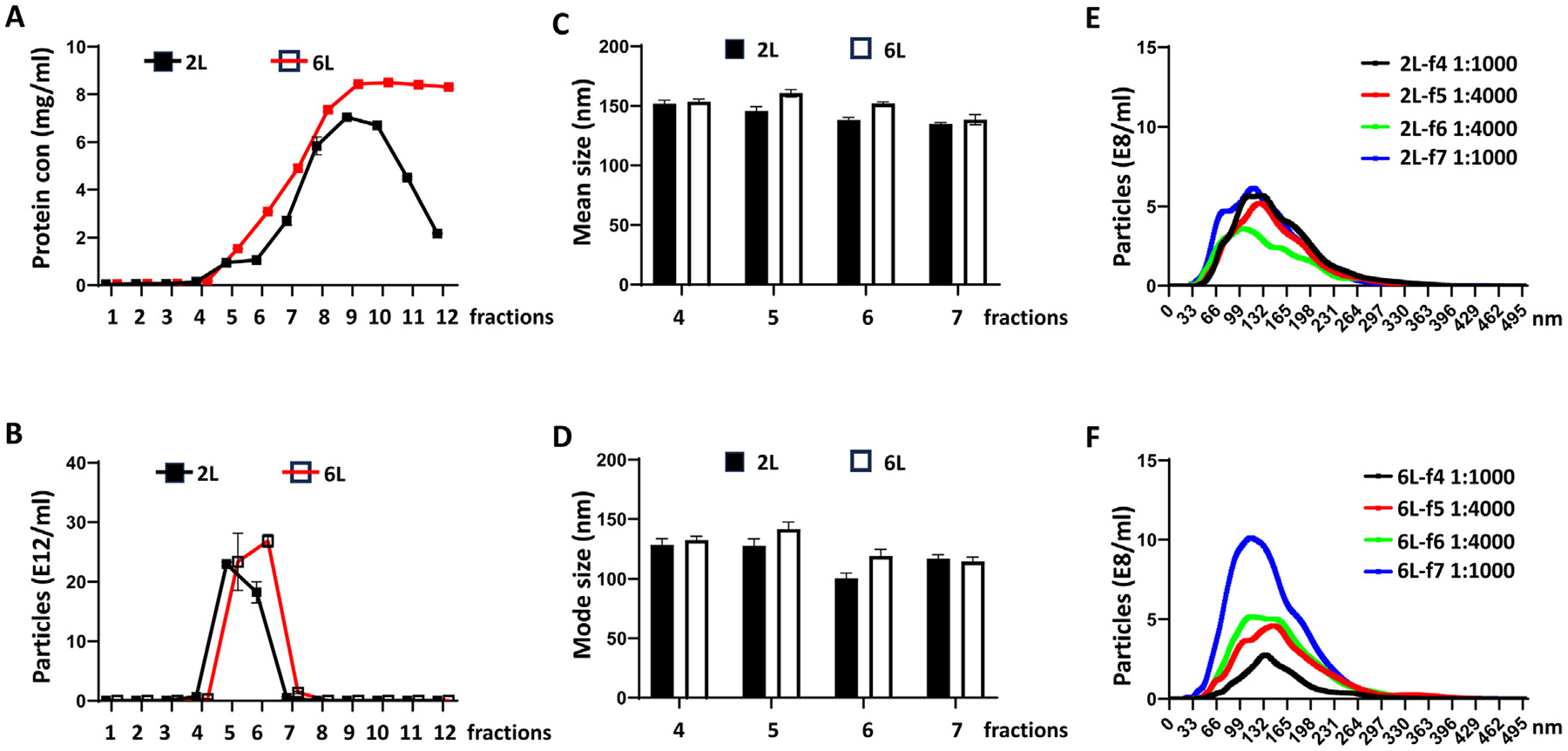
Size-exclusive chromatography (SEC)–based isolation of Wharton’s jelly-derived mesenchymal stromal cell (WJMSC) small extracellular vesicles (sEVs). Tangential flow filtration (TFF)–concentrated medium was used to isolate the EV populations using size exclusion chromatography (SEC). (A) The total protein concentrations of different SEC fractions. (B) The averaged WJMSC sEV counts from (A) measured by nanoparticle tracking analysis (NTA). (C,D) The WJMSC sEV sizes were measured by NTA. Mean sizes (nm, C) and mode sizes (nm, D). (E,F) The averaged size distributions of WJMSC sEV isolations; 2 L (E) and 6 L (F). Values on graphs represent means ± SEM, n = 3 individual experiments (A–D).

To isolate the WJMSC-derived sEVs, we further applied SEC to perform the in-depth purification using the retentate (concentrated) samples (Figure 1B). We found that WJMSC sEVs can be collected from the fourth and seventh elution fractions. For example, from the 2-L batch, 0.7 ± 0.03, 23 ± 0.4, 18 ± 1.8, and 0.5 ± 0.2 × 10^12^ particles per milliliter were detected by NTA in the elution fractions #4, #5, #6, and #7, respectively (Figure 3B). Similar results were obtained from the 6-L batch. We observed 0.3 ± 0.04 and 1.4 ± 0.2 × 10^12^ particles per milliliter from the elution fractions #4 to #7, respectively, from the 6-L batch. The maximal yield of particles appeared in either fraction #5 or fraction #6 for both the 2-L and 6-L manufacturing batches. Further calculation showed that 61 mg (2.1 × 1013/mg) and 231 mg (1.1 × 1014/mg) of WJMSC-derived sEVs were obtained from 2-L and 6-L batches. However, we noticed that the yield of particles was not directly correlated to the total protein level, as shown in Figure 3A. As an example, the total protein level from fractions #6 to #7 of the 2-L batch was decreased by 61% although their particles increased by 92-fold. This observation indicates that our 300-KD cutoff and diafiltration from the TFF manufacturing did not remove all soluble protein from the particles.

**Figure 4.**
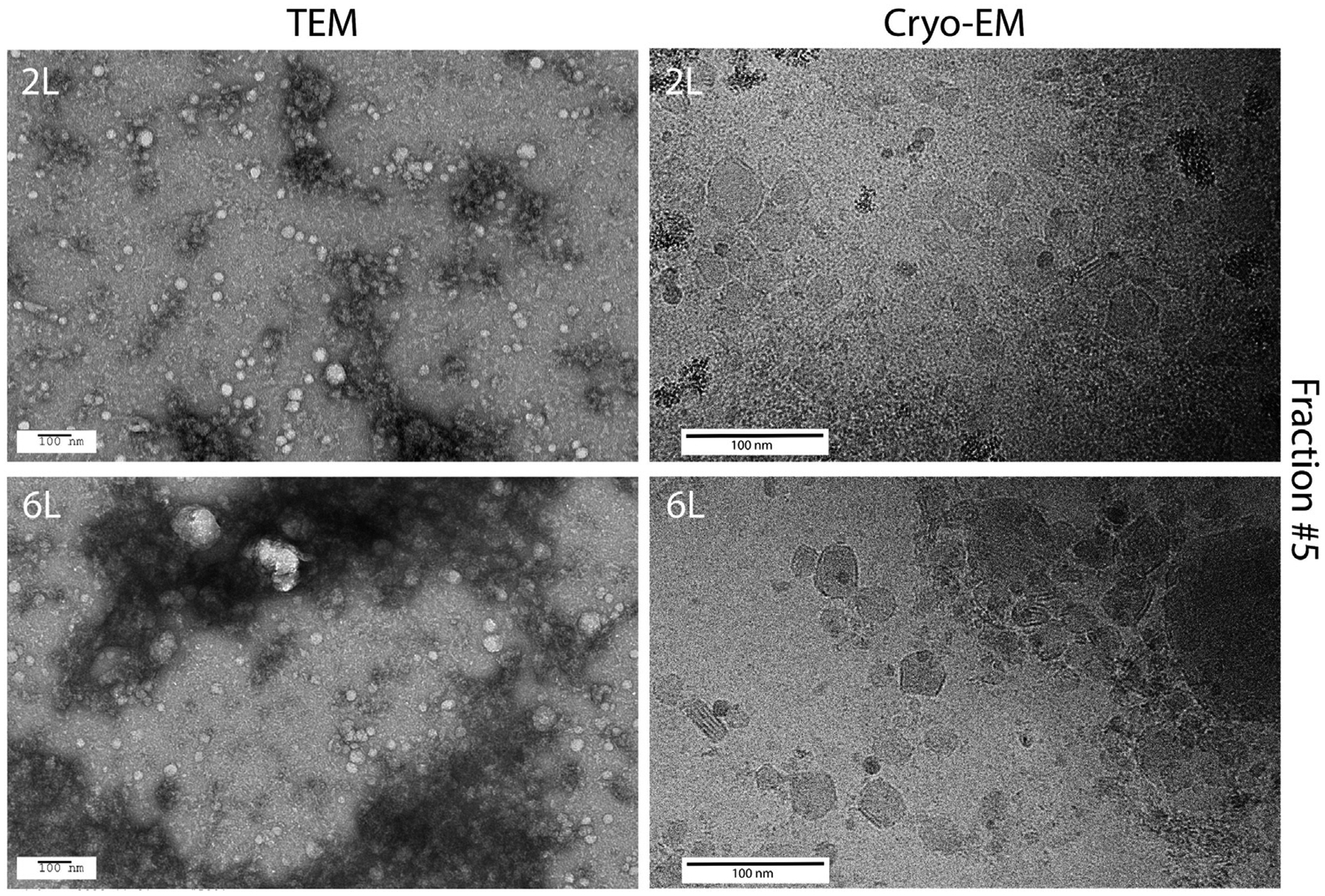
Morphology of purified Wharton’s jelly-derived mesenchymal stromal cell (WJMSC) small extracellular vesicles (sEVs). Representative transmission electron microscopy (TEM, *left*) and cryogenic electron microscopy (Cryo-EM, *right*) imaging of therapeutic WJMSC sEVs isolated by size exclusion chromatography fraction #5 (*top*, 2 L; *bottom*, 6 L). Scale bars, 100 nm.

We further examined the particle sizes from fractions #4 to #7 and found that their mean sizes ranged from 135 ± 1.3 nm to 154 ± 2 nm (Figure 3C–F). These small extracellular vesicles demonstrated the typical sEVs morphology (lipid bilayer membrane) and structural integrity visualized by transmission electron microscopy (TEM) and cryogenic electron microscopy (cryo-EM) as shown in Figure 4. Consistently, our WES results showed that sEVs biomarkers CD9 and CD81 were enriched on the nanoparticles isolated by SEC (Figure 6A and Supplemental Figure 5). Our WES results also demonstrated that CD73, a stem cell biomarker, was highly enriched in sEVs, and calnexin, an endoplasmic reticulum (ER) biomarker was not detected on these nanoparticles (Figure 6B, and Supplemental Figure 5). Importantly, we noticed an obvious separation of albumin protein between fraction #5 and fraction #7. To exclude the potential contamination from soluble proteins, we collected the WJMSC sEVs mainly from fractions #5 and #6. We thus estimated that the total yield of WJMSC-derived sEVs from the 2-L and 6-L batches can be between 2.5 and 5 × 10^15^ (Table 2). All purified WJMSC sEVs have passed the validations of endotoxin, mycoplasma, and sterility according to the GMP-compliant regulations (Table 3). Therefore, we demonstrated a combined platform to manufacture large-scale WJMSC sEVs with high yield and purity.

### Evaluation of immunosuppressive WJMSC-derived sEVs

**Figure 5.**
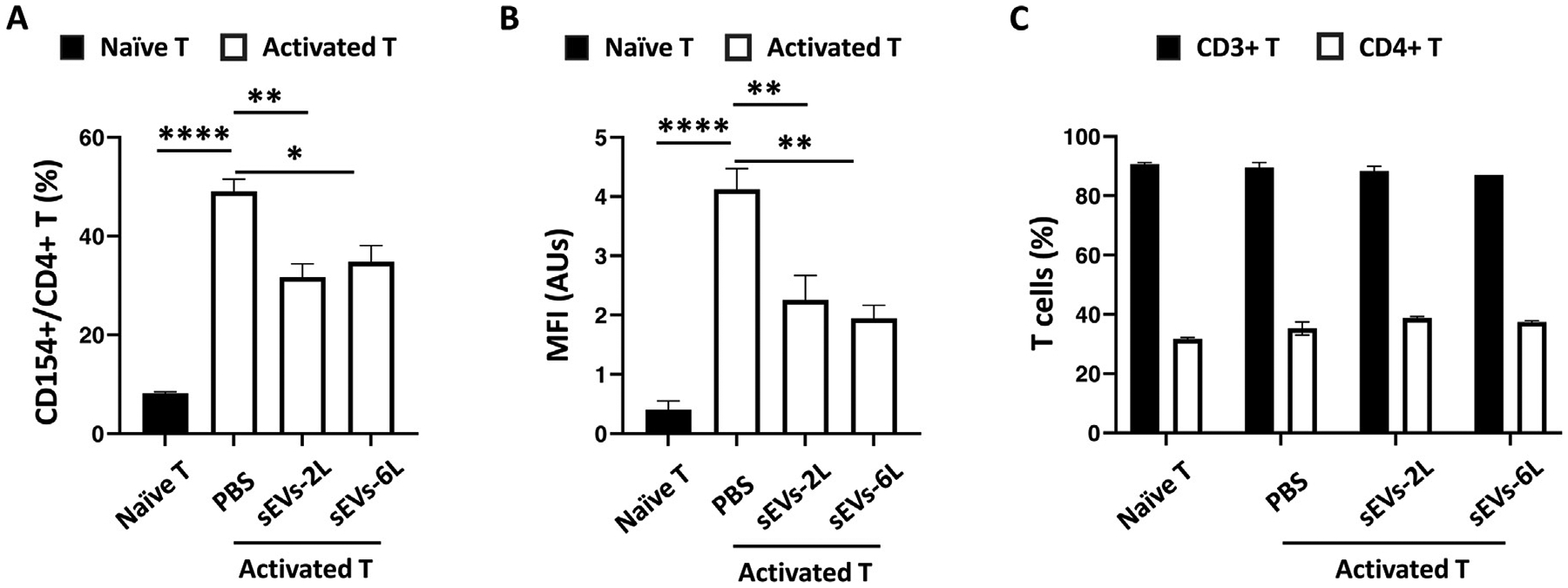
Inhibitory Wharton’s jelly-derived mesenchymal stromal cell (WJMSC) small extracellular vesicle (sEV) exhausted T-cell activation in vitro. (A,B) Activated CD4^+^ T cells were inducted in vitro by CD3/CD28 Dynabeads (Thermo Fisher Scientific, IL, USA) and visualized by anti-human CD154 antibody using flow cytometry. Cell counts (A) and mean fluorescent intensity (B) from the CD154^+^/CD4^+^ T cells. Values on graphs represent means ± SEM, n = 3–4 treatments. *P < 0.05, **P < 0.01, ****P < 0.001. (C) Total CD3^+^ and CD4^+^ T cells from (A) and (B). 8 × 10^3^ particles/cell of WJMSC sEVs (sEVs 2 L and sEVs 6 L) were applied to inhibit T cell activation (A,B).

To investigate whether WJMSC-derived sEVs maintained their bioactivity after manufacture, we utilized naïve healthy donor PBMCs that were initially stimulated by CD3/CD28 Dynabeads *in vitro*. Then, the activated CD4^+^ T cells were detected by CD154 expression using flow cytometry. As shown in Figure 5A and Supplemental Figure 4, activated PBMCs in the control (PBS) group had significantly higher CD4^+^/CD154^+^ T cells (49% ± 3%) compared with only 8% ± 0.3% of CD4^+^/CD154^+^ cells from the naïve group. Consistently, mean fluorescent intensity (MFI) of CD154 demonstrated a 10-fold enhancement of fluorescent intensity compared with the naïve CD4^+^ T cells (Figure 5B). Second, we tested the immunosuppressive potential of WJMSC sEVs on activated PBMCs. After treatment with WJMSC sEVs at a concentration of 8 × 10^3^ particles/cell (SEC fraction #5), we observed less activation of CD4^+^ T cells with CD4^+^/CD154^+^ 32% ± 10% from the 2-L batch and 33% ± 19% from the 6-L batch in the sEV-treated groups compared with the control (PBS) group (Figure 5A). Similar results were obtained by analyzing the MFI of CD154 expression (Figure 5B). We found that CD154 mean fluorescent intensity was decreased by 48 ± 10%~51 ± 3% in the treated group compared with the PBS control. As an intra-control, we noticed that the total numbers of live CD3^+^ and CD4^+^ T cells were not changed in both treated and untreated activated CD4^+^ T cells (Figure 5C). These observations support that manufactured WJMSC sEVs still maintain their immunosuppressive activity.

**Figure 6.**
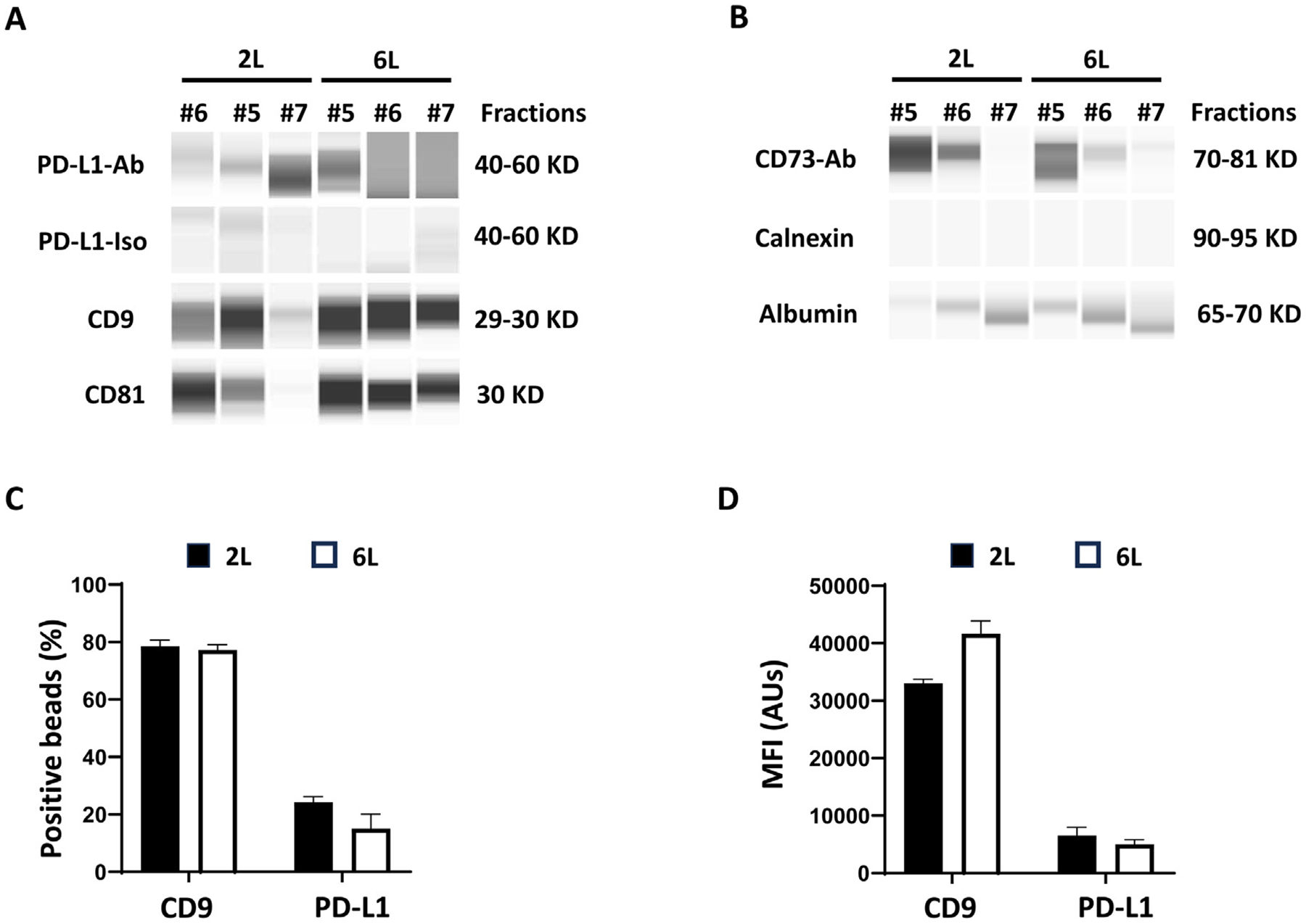
Detection of PD-L1 on inhibitory Wharton’s jelly-derived mesenchymal stromal cell (WJMSC) small extracellular vesicles (sEVs). (A) PD-L1 protein was detected on the WJMSC sEV lysates of size-exclusive chromatography (SEC) fractions #5 to #7 using automated western assay (WES). Ab, antibody, and Iso, isotype. (B, C) sEVs biomarker CD9 and checkpoint protein PD-L1 were detected by flow cytometry on the WJMSC-derived sEVs from SEC fraction #5 after being captured with CD9 beads. All data were normalized by the isotypes and values on graphs represent means ± SEM, n = 3 measurements. Cell counts (A) and MFI, mean fluorescent intensity (B).

Our previous studies suggested that PD-L1 on WJMSC-associated sEVs is associated with immunosuppression [11]. As shown in Figure 6 and Supplementary Figure 5, our WES results showed a potential enrichment of PD-L1 on the WJMSC sEVs after combined TFF and SEC manufacturing. To confirm this observation, we applied flow cytometry to detect both PD-L1 and CD9 on WJMSC sEVs that were captured by CD9-positive beads. Our results demonstrated that 77 ± 2%~79 ± 2% beads were CD9-positive, indicating a successful capture of CD9-positive WJMSC sEVs on these beads (Figure 6B). Among these sEVs, we further observed 15 ± 5~24 ± 2% of captured beads showing PD-L1 positive, further confirmed by analyzing their MFI (Figure 6). These findings support that WJMSC-derived sEVs maintain not only their immunosuppressive activity but also the inhibitory immune ligands (PD-L1) after combined TFF and SEC manufacturing.

## Discussion

The development of therapeutic WJMSC-derived extracellular vesicles for human immune and degenerative diseases depends on the successful manufacturing of high-quality sEVs. To achieve this long-term goal, we have successfully established a protocol to grow WJMSCs and obtain their conditioned medium as a source of clinical-grade sEVs. Compared with previous large-scale production methods [33,34], our sEV protocol combined with both TFF and SEC. Importantly, the approach was optimized for cell growth in non-serum WJMSC culture medium to help meet scalable industry-level manufacturing requirements for the isolation of potential therapeutic sEVs.

Our previous study applied therapeutic WJMSCs (MSCTC-0010) generated from the platform utilized to treat aGVHD patients in a clinical trial [10]. Treatment of patients with aGVHD with low- or high-dose WJMSCs was safe: the infusion was well tolerated, and no severe treatment-related adverse events or ectopic tissue formation was observed. A clinical improvement was seen in about 70% of patients, with 4 of 10 showing a complete response after MSCTC-0010 infusions. We demonstrated previously that these patients had increased sEV PD-L1 levels in the blood post-MSCTC-0010 infusion and potentially related to the clinical activity of therapeutic WJMSCs [11].

We employed a manufacturing platform for WJMSC-derived sEV production, including TFF for initial medium concentration, and SEC for comprehensive EV purification from soluble protein. An updated quality control (QC) has been therefore suggested for EV manufacture and validation (Table 3). We manufactured WJMSC sEVs from 2 pilot batches of WJMSC culture medium from an umbilical cord, including a small volume (2 L) and a large volume for scaling up (6 L). These 2 batches have been systematically and comparatively tested. We successfully obtained ~61 mg (2.1 × 10^13^/mg) and ~231 mg (1.1 × 10^14^/mg) of WJMSC-derived sEVs from 2 batches (2 L and 6 L) of WJMSC culture medium, respectively. These manufactured WJMSC sEVs demonstrated an intact structure and stable inhibitory effects on T-cell activation *in vitro*. More importantly, we observed that about 15% to 24% of WJMSC sEVs were PD-L1 positive, representing a specific therapeutic subpopulation.

During TFF-based concentration, a 300-KD cutoff was chosen for the polyethersulfone ultrafiltration cassette to separate the sEVs from other soluble proteins. Although the predicted molecular weights of sEVs are <100 KD, we did not find significant WJMSC sEVs from the permeate part of the TFF-based concentration. This finding indicates that the actual molecular weights for WJMSC-derived EVs may be >100 KD or even >300 KD. Both 300-KD and 500-KD cutoffs have been reported for TFF-based sEV isolation and purification. In this study, we applied 1 cycle of concentration and diafiltration to initially concentrate the nanoparticles starting from 2 L and 6 L of WJMSC media (Supplementary Table 1). Compared with the original input medium, we observed a significant concentration efficacy of nanoparticles from both batches after TFF to a final 17- to 36-fold concentration. Under current TFF conditions, 40% to 48% of nanoparticles were successfully recovered, and more than 90% of proteins were removed. In addition, we noticed differences in particle total numbers between 2 L and 6 L due to a higher concentration ratio in the 6-L batch. According to our current TFF parameters, we estimated that the Sartoflow Smart system plus the maximal 0.14 m^2^ membrane could process more than 12.5 L of input medium, demonstrating very efficient processing for large-scale manufacturing of therapeutic sEVs. To further purify WJMSC sEVs, we applied SEC to isolate the sEV particles from other components concentrated in the retentate part from the TFF-based concentration. Like classical morphology of sEVs, isolated WJMSC sEVs by the method developed demonstrated 135- to 154-nm mean sizes and 100- to 134-nm mode sizes. However, we noticed that 5% human platelet lysate potentially contributed to about 20% of particles in the medium. To remove these exogenous sEVs acquired during the upstream process of the WJMSC growth stage, we suggest further optimization of both the WJMSC culture and sEV manufacturing in the future. Previously, we have centrifuged fetal bovine serum 100 kg overnight before adding to the basal medium; we would need to test this approach with platelet lysate for large-scale manufacturing [11]. This premanufacturing step will ensure the removal of most nanoparticles from the platelet lysate supplement. Platelet-free or serum-free opti-MEM medium has been used to collect sEVs prior to EV manufacturing [35]. More importantly, as the only truly designed EV-free medium, recent studies suggested that chemically defined medium formulation may have the advantage of improving the purity of WJMSC-derived sEVs without other potential EV contaminations [22,23].

It has been reported that the immune checkpoint protein ligand PD-L1 was enriched on the WJMSC-derived sEVs compared with their parental WJMSCs [11]. Genetic disruption of the *PDL1* gene in WJMSCs knocked out the PD-L1 protein from the WJMSC-derived sEVs. PD-L1-deficient WJMSC sEVs lost their immunosuppressive capacity on activated T cells, indicating an essential function of WJMSC sEVs during immune modulation [11]. Comparing 2 different manufacturing approaches, we found that sEVs purified from both batches maintain their potential to inhibit T-cell activation. We further examined the PD-L1 protein on those sEVs purified from different manufacturing processes. Similar results were obtained indicating that 15% to 24% of sEVs are PD-L1 positive for both manufacturing processes. As a control, we noticed that 77% to 79% of sEVs are CD9 positive. Our observations support that at least 15% to 24% sEVs are inhibitory sEVs, and different manufacturing processes did not directly impact the enrichment of PD-L1 on sEVs. This is in line with our previous results and the PD-L1+ sEVs are derived from WJMSC and less likely to be contaminated by platelet lysate nanoparticles. These results indicate that checkpoint PD-L1 is a potential biomarker for validating immunosuppressive sEVs.

Quality control (QC) for biological manufacturing is important to ensure the safety and efficacy of MSC-based products for patients [17,36]. Although MSC manufacturing QCs have been established, sEV manufacturing QC has not reached a broad consensus [17,37,38]. To match the combined manufacturing platforms established in the current study, QC updated from the previous WJMSC clinical trial was first used to examine the pathogens in the WJMSC culture medium under the upstream GMP-compliant production (Table 1) [10]. Second, a more complex system was used to validate the WJMSC sEVs and relevant downstream EV manufacturing (Table 3). For example, pathogen tests were conducted by examining endotoxin, mycoplasma, and bacteria; sEVs’ morphology and their structure integrity were evaluated by TEM and cryo-EM. To identify WJMSC sEVs, we applied WES or bead-based flow cytometry to analyze the sEV biomarkers CD9 and CD81 on their membrane surface. We suggest that PD-L1 could be an important biomarker to quantify a specific inhibitory sEV subpopulation enriched by checkpoint PD-L1 through WES or bead-based flow cytometry. More importantly, WJMSC sEV bioactivity can be determined by *in vitro* inhibition of CD4^+^ T-cell activation mediated by T-cell receptors. In the current study, we established a systematic QC process to monitor the large-scale production of therapeutic EVs at different stages and validate their therapeutic qualities. However, we also realize that human clinical trials are essential to validate their safety and efficacy.

In summary, our study demonstrates a reliable large-scale production for immunosuppressive WJMSC-derived sEVs and QC systems for further GMP-compliant manufacturing. Our exploration will facilitate the industrial standard for developing and producing clinical-grade therapeutic WJMSC sEVs for human clinical trials in the future.

## Supplementary Material

1

Supplementary material associated with this article can be found in the online version at doi:10.1016/j.jcyt.2025.06.003.

## Figures and Tables

**Table 1:** Validation of GMP-compliant WJMSC culture medium.

Analytical Test	Methods	Acceptance criteria	Results
Endotoxin	Bacterial endotoxin USP <85>	≤5 EU/mL	≤0.05 EU/mL
Mycoplasma	Polymerase chain reaction	Not detected	Not detected
Sterility	Microbiological control of cellular product USP <71>	No growth for 14 days	No bacterial or fungal growth for 14 days

**Table 2:** Summary of scalable manufactures of WJMSC sEVs.

	Low-volume manufacture	High-volume manufacture
	TFF concentration	
Input volume, IV (L)	2L	6L
Final volume, FV (L)	61 mL	100 mL
Concentration ratio (IV vs. FV)	1:33	1:60
	SEC purification per fraction	
Particle counts, Pct (×10^12^/mL)	23	23
Protein concentration, Pcon (mg/mL)	0.9	1.5
Purity index (Pct vs. Pcon)	26	15
Total predicted WJMSC-sEVs yield	~2.5 × 10^15^	~5 × 10^12^

SEC, size-exclusive chromatography; sEVs, small extracellular vesicles; TFF, tangential flow filtration; WJMSC, Wharton’s jelly-derived mesenchymal stromal cell.

**Table 3:** The validation of purified inhibitory WJMSC sEVs.

Analytical test	Methods	Acceptance criteria
Particle protein	BCA	NA
Particle number and size	NTA	NA
Immunosuppression	CD3/CD28 antibodies induced T-cell activation	20%
Endotoxin	Bacterial endotoxin USP <85>	≤ 5 EU/mL
Mycoplasma	PCR	Not detected
Sterility	Microbiological control of cellular product USP <71>	No growth for 14 days
Tetraspanin proteins	Flow cytometry and WES	CD9 or CD81 positive
CD9 or CD81 positive	Flow cytometry and WES	PD-L1 positive
Morphology	TEM or Cryo TEM	Normal structure

BCA, bicinchoninic acid; Cryo TEM, cryogenic transmission electron microscopy; NA, non applicable; NTA, nanoparticle tracking analysis; PCR, Polymerase chain reaction; sEVs, small extracellular vesicles; TEM, transmission electron microscopy; WES, automated western assay; WJMSC, Wharton’s jelly-derived mesenchymal stromal cell.
