# Supplementary material for: Large-scale manufacturing of immunosuppressive extracellular vesicles for human clinical trials
Source: Cytotherapy. Author manuscript; Available in PMC 2026 Jun 15. (PMC13267737; doi:10.1016/j.jcyt.2025.06.003)
Supplement: 1 [file NIHMS2183417-supplement-1.docx]

**Supplemental Figure 1. Particle numbers and sizes from TFF-concentrated samples.** (**A**) Particle and mean size distribution of 2L- and 6L- concentrated medium (CM). Input CM and retentate were diluted into EV-free PBS by 1/500 and 1/10,000, respectively for the NTA analysis. Input CM, original conditional medium (black and green curves). Retentate, concentrated medium after TFF (red and purple curves). Values on graphs represent the dilution ratio. (**B**) Mode size (nm, left) and mean size (nm, right) from **A**. Values on graphs represent means ± sem, n = 3 measurements.

**Supplemental Figure 2. TFF-based concentration of basal medium without WJMS culture.** Basal medium (2L) and conditioned medium (2L) were individually concentrated by tangential flow filtration (TFF) with a 300KD cutoff. (**A**) Summary of retentate and permeate volumes after the TFF. (**B**) The total protein measurements of input CM (original medium), retentate, and permeate were analyzed by BCA. n = 3 measurements. (**C**) The total numbers from (B) were measured by nanoparticle tracking analysis (NTA). n = 5 measurements. (**D**-**E**) Mode size (nm, left) and mean size (nm, right) from **A**. n = 5 measurements. Values on graphs represent mean ± sem (B-E).

**Supplemental Figure 3. The protein level of type I collagen during was examined by ELISA**. (**A**) Samples from Tangential flow filtration (TFF) include original medium (input CM), retentate, and permeate. (**B**) 12 elution fractions are generated through size exclusion chromatography (SEC). Values on graphs represent means ± sem, n = 3 individual measurements (**A** and **B**).

**Supplemental Figure 4. Representative flow charts of activated T cells blocked by inhibitory WJMSC sEV.** (**A**) CD3^+^ T cells. (**B**) CD4^+^ and CD8^+^ T cells. (**C**) CD154^+^/CD4^+^ T cells. Naïve T cells were activated by CD3/CD28 Dynabeads.

**Supplemental Figure 5. Semi-quantitative analysis of WES.** The WES was conducted using protein lysates from SEC-purified WJMSC sEVs. (**A**) CD81; (**B**) CD9; (**C**) PD-L1-Ab, Antibody; (**D**) PD-L1-Iso, Isotype, Isotype; (**E**) CD73; (**F**) Albumin; (**G**) Calnexin.

**Supplemental Figure 6. Representative flow charts demonstrating EV-associated biomarker CD9 and checkpoint PD-L1 on the WJMSC sEVs (fraction #5 from SEC purification).** (**A**) PD-L1 and (**B**) CD9. WJMSC sEVs from 2L (top) and 6L(bottom).

| Parameters | 2L | 6L |
| --- | --- | --- |
| Molecule reference | sEVs | sEVs |
| MWCO | 300KD | 300KD |
| Membrane Polymer | PES | PES |
| Order numbers | 3M81467902E | 3M51467901E |
| Membrane area (m^2^) | 0.02 | 0.14 |
| Start volume (mL) | 1951 | 5987 |
| Initial (optimum) concentration factor DF (X) | 33 | 60 |
| Diavolumes number | ~8.5 | ~9.0 |
| Diafiltration buffer | Exosome-free PBS | Exosome-free PBS |
| Final/overall concentration factor (X) | 61 | 100 |
| Optimum permeate flux via optimization (LMH) | ~57 | ~57 |
| Average permeate flux throughout entire process (LMH) | ~35 | ~35 |

**Supplemental Table 1. The parameters of concentration and diafiltration of tangential flow filtration.**
